# An investigation of the equine infectious disease threat represented by the presence of donkeys at mixed equestrian events in Ireland

**DOI:** 10.1186/s13620-015-0041-6

**Published:** 2015-06-12

**Authors:** Sarah Finney, Joseph A. Collins, Vivienne Duggan

**Affiliations:** School of Veterinary Medicine, University College Dublin, Dublin, Ireland; Deerpark Veterinary Services, Castlelyons, Co Cork Ireland

## Abstract

**Background:**

The number of abandoned or otherwise neglected donkeys has significantly increased in Ireland in the recent past. The real or perceived capacity of the donkey to act as a reservoir of equine infectious disease, and thus pose an increased risk of disease transmission to horses and ponies, may be a factor in this increased abandonment and neglect. The authors here report on a field study exploring the infectious disease transmission threat the donkey poses to the general equine industry in Ireland through an examination of biosecurity standards and the views of horse and donkey exhibitors at nine mixed equestrian events in 2014. Quantitative information was gathered via the organising committee (if any) and through an examination of facilities and procedures. Qualitative information was gathered using a semi-structured questionnaire to ascertain the view of exhibitors regarding the keeping of donkeys and any infectious disease transmission risks posed.

**Results:**

At eight of nine events visited there were no entrance controls, no veterinary examinations, no enforcement of legislation regarding equine identification and equine premises registration and no isolation facilities on site for equids. Contact between donkeys and other equids was largely uncontrolled. Exhibitors had travelled from abroad to one event. Exhibitors generally opined that they did not perceive the donkey to represent any additional infectious disease transmission threat above that posed by other equids; there was however a general sense that donkeys were less well regarded for other reasons including nuisance and uselessness.

**Conclusions:**

When biosecurity controls are not in place (or enforced) to actually check passports, verify identification and equine premises registration, mixed equestrian events may unwittingly act as the mechanism of spread of endemic and potentially more seriously exotic equine infectious disease. Donkeys were not generally considered by equine exhibitors at mixed events in Ireland to represent a heightened reservoir of disease or to pose an increased risk of transmission of contagious disease suggesting that other factors should be considered more important when studying the incidence of abandonment and neglect.

## Background

In the recent past, in particular with the economic decline seen in Ireland since 2007, the number of abandoned or otherwise neglected donkeys has significantly increased. By way of example, over 1400 donkeys are currently in the care and stewardship of the Donkey Sanctuary, based in Co. Cork (N Carton, 2014 Donkey Sanctuary Ireland personal observation): approximately 1100 on managed farms and some 350 spread among foster homes on the island of Ireland mostly as bonded pairs. This is despite sending an average of over 270 donkeys to the Donkey Sanctuary in GB annually in recent years from the 32 counties of Ireland. Comparable figures for Ireland for 2008 were fewer than 400 donkeys on managed farms and 485 in foster care. The Donkey Sanctuary (Ireland) acquired over 400 new donkeys requiring care in 2013 and the seemingly ever-growing population of neglected, abandoned and relinquished donkeys in Ireland has become a growing cause for concern and discussion vis a vis their origin, why they are bred or kept, and in particular why they are being relinquished in large numbers in recent years.

The donkey’s capacity to act or perception as acting as a reservoir of equine infectious disease may be of relevance to this discussion amongst those in the general equine industry, particularly where equine husbandry and care standards are low. Collins et al [1] reported on threats to equine welfare in Ireland highlighting (among other issues) the potential for the transmission of contagious disease at equestrian events where there is little enforcement of identification of equidae legislation and variable, often poor, standards of biosecurity. Subsequently demographic data regarding the numbers of unwanted equidae including donkeys in Ireland has been compiled [2]. There is a link between the incidence of unwanted, poor value equidae, the neglect of these animals’ health needs (including vaccination and deworming) and the threat of the spread of contagious disease at equine events where good biosecurity standards and traceability are lacking. The risk of transmission of contagious disease at unregulated equine events is of immediate relevance with regard to the spread of endemic viral, bacterial, parasitic and fungal infections but would also be of critical concern in the instance of an outbreak of an exotic or listed disease which would prove a threat to the entire equine industry.

While donkeys show similar clinical signs as horses of equine herpesvirus, equine influenza virus and Strangles (*Streptococcus equi* var *equi*) infection [3, 4] donkeys are known to be less severely affected clinically by diseases such as Equine Viral Arteritis (EVA) [4] and asymptomatic carriers of other endemic diseases such as lungworm (Dictyocaulus arnfeldi) [5]. Of further concern is the subclinical or asymptomatic nature of infection of donkeys with critical exotic diseases including Equine Infectious Anemia (EIA), African Horse sickness (AHS), Piroplasmosis (Theileria equi) and Trypanosoma equiperidum (the agent of Dourine) [4, 6, 7]. For EIA, it is of note that when testing using the AGID or Coggins test, antibodies for EIA show up later in donkeys then in horses [4]. For AHS it is of note that even when vaccinated, donkeys may act as a reservoir for disease [4]. A recent qualitative risk assessment carried out by the Irish Department of Agriculture, Food and the Marine (DAFM) reported the risk of the incursion of African Horse Sickness into the Republic of Ireland by a legally imported equid to be ‘very low’; however, the risk associated with illegal importation of equidae or their products was reported as ‘very difficult to quantify’ [8]. Continued vigilance in Ireland with regard to arthropod-borne (currently exotic) diseases such as AHS is urged [9].

The authors here report on a study employing a combination of quantitative and qualitative research methodologies to explore the threat the donkey poses as a reservoir of infectious disease to the general equine industry in Ireland through an examination at mixed equestrian events of:

- Biosecurity standards, via an assessment of
  - requirements for entry to event classes and entrance to the event premises;
  - compliance with relevant national legislation related to the identification and keeping of equidae; of biosecurity procedures in evidence;
  - observation of the clinical signs of equine ill-health;
  - enquiry as to the place of origin of the exhibitors of equines.
- The perception among the exhibitors of donkeys regarding
  - donkeys as a significant biosecurity risk in the transmission of contagious equine disease to horses and ponies;
  - their experience of how donkeys and donkey keepers (DKs) are perceived.
- The perception among equestrian exhibitors in general regarding
  - the problems posed by the keeping of donkeys viz a viz other equidae;
  - their view of donkeys and DKs.

If donkeys are perceived to be a significant reservoir for equine contagious disease, or other significant problem by the general leisure and pleasure horse industry in Ireland, this perception may serve to increase the risk of abandonment and reduce the desire to foster thus driving up the numbers (needing to be) kept by donkey sanctuary organisations.

## Methods

A schedule of equestrian events held during the summer months in Ireland was developed. Events to be visited were selected based on the following criteria:

- Historical attendance figures of donkeys
- The spectrum of horse/pony/donkey classes scheduled
- Geographical location and spread around the island of Ireland
- Occurrence during the summer months of 2014

Nine events throughout Ireland were subsequently visited by at least one, often two of the authors to gather information. They are named below in alphabetical order, but listed as events 1 to 9 in the results section in the order in which they were attended:

- Cahirmee Horse Fair, Co Cork
- Castlewellan Agricultural Show, Co Down
- Clonmel Show. Co Tipperary
- Cultra Donkey Day, Co Down
- Dublin Horse Show, Ballsbridge, Dublin
- Gort & District Show, Co Wexford
- Kilmancanogue Horse Show, Co Wicklow
- Mullingar Agricultural Show, Co Westmeath
- Newcastle West Show, Co Limerick

### Biosecurity standards

A representative of the event organizers was contacted either in advance by telephone and/or email and/or at the event and asked to describe controls relevant to equine biosecurity:

- Pre-registration and/or onsite registration for horse/pony and donkey classes
- Event entry requirements—identification (passports, microchips), vaccinations etc.
- Entrance/exit control for horses/ponies/donkeys, and compared to other livestock if these present
- Veterinary personnel onsite and/or on call
- Territory of origin of exhibitors of horses, ponies and donkeys

The above were assessed during the visit by the researcher(s) to the event as well as an evaluation of:

- The numbers of donkeys and donkey exhibitors; how many donkeys they kept and whether they kept them with horses
- Contact at the event between horses and donkeys
- Sharing of equipment that might act as disease fomites
- Overall equine health/welfare standards on visual inspection
- Donkey Body Condition Scores (BCS) on a scale of 1 = poor to 5 = obese [4]

### Donkey exhibitor views

Donkey exhibitors present at each event were approached and interviewed by one of the authors. The topics for discussion were set in the form of a semi-structured questionnaire as follows; however the interviewee determined the breadth and depth of material covered:

- General background regarding their interest in donkeys
- Their awareness and compliance with the requirements for donkey identification or any other legislative provisions
- Their reasons why they bred and/or kept donkeys
- Where they procured donkeys from if they didn’t exclusively breed them
- Their view of the health needs of donkeys including vaccination and de-worming
- Whether they experienced objections or prejudice by horse owners living near them or while attending mixed equestrian events

In general open questions were used to encourage discussion and engagement; closed questions were used to clarify particular items.

### Equine exhibitor views

Horse/pony keepers (HKs) greatly outnumbered donkey keepers (DKs). Both were questioned on a random, ad hoc basis as the running of the event and classes permitted. They were asked about:

- General background equestrian matters
- Their perception of donkeys as a source of diseases potentially contagious to horses
- If a HK, whether they experienced or held a prejudice against donkeys and/or those who kept them (DKs), and whether they objected to donkeys being kept near them or attending mixed equestrian events
- If a DK, whether they experienced prejudice against donkeys and DKs
- Problems with the keeping of donkeys
- The value of donkeys

These views were collated and analysed for global, organizing and basic themes [10].

## Results

### Biosecurity standards: the findings are summarized in Table 1

**Table 1:** A summary of quantitative data gathered at mixed equestrian events in Ireland. The nine events are listed in the order in which they were visited in the summer months of 2014

Event No	Event requirements	Entrance/On-site controls	Veterinary services	Number of donkeys and donkey exhibitors	Contact between horses and donkeys	Any health issues witnessed
1	Register on-site for showjumping, pre-register for showing classes	no records checked at gate, passports not checked on-site	veterinarians on hand	10, same owner runs derby	donkeys stabled separately and donkey derby in separate ring to horse/pony classes	no
2	Mainly pre-registration, only a few last minute on-site registrations	no records checked at gate, passports to be shown to ring judge	no veterinarians on premise	6 donkeys, 2 families owned	horses walked freely next to donkeys	no
3	Both pre- & on-site registration. Passport & premises registration required to register	no records checked at gate	veterinarian on site, on call for issues	18 donkeys, various owners	Show rings adjacent to each other for horses and donkeys	no
4	Both pre- & on-site registration. Passport & premises registration required to register horses, not required for donkeys	no records checked at gate	veterinarian on-site, another on call for issues	5 donkeys - 2 different owners	donkeys kept in small pens next to mini horses	skin lesions, poor coats, over-weight, over-grown hooves, nasal discharge, scour
5	No registration requirements.	No records checked at the multiple entry points	none	20 donkeys, owners not reliably in associated attendance	No controls on mixing of species	Donkeys in reasonable body condition but unkempt
6	Both pre- & on-site registration. No passport or premises registration required	no records checked at gate	no veterinarian on-site, but one on call	3 donkeys - 2 owners	donkeys shown in separate ring	no
7	No registration requirements; all members of The Donkey Breed Society	no records checked at gate	none	15 donkeys	no horses at event	no
8	All equines pre-registered. Passport required to register. Vaccination against equine influenza required.	All records checked at single entry/exit	Multiple veterinarians presence	24 donkeys	Contact between species once on event premises	no
9	Both pre- & on-site registration. Passport & equine premises registration required to register	no records checked at gate	none	2 donkeys	donkeys kept in same area as horses	no

#### Registration requirements

At eight out of nine events, pre-registration was required of the entrants to at least some equestrian classes; five events allowed on-site registration to selected classes e.g. showing classes but not showjumping classes; one event did not have any entry requirements of any type for horses, ponies or donkeys.

#### Biosecurity procedures in evidence

Entrance controls at eight out of nine events were non-existent: anyone with a horsebox or livestock trailer was let into the horsebox car park and subsequently onto the show ground. At these eight events, no veterinary examinations were conducted on animals upon arrival; at five of these eight events, veterinary personnel were present on-site or available on call. None of these eight had isolation facilities; neither were there hand washing or boot dipping facilities.

**Fig. 1:**
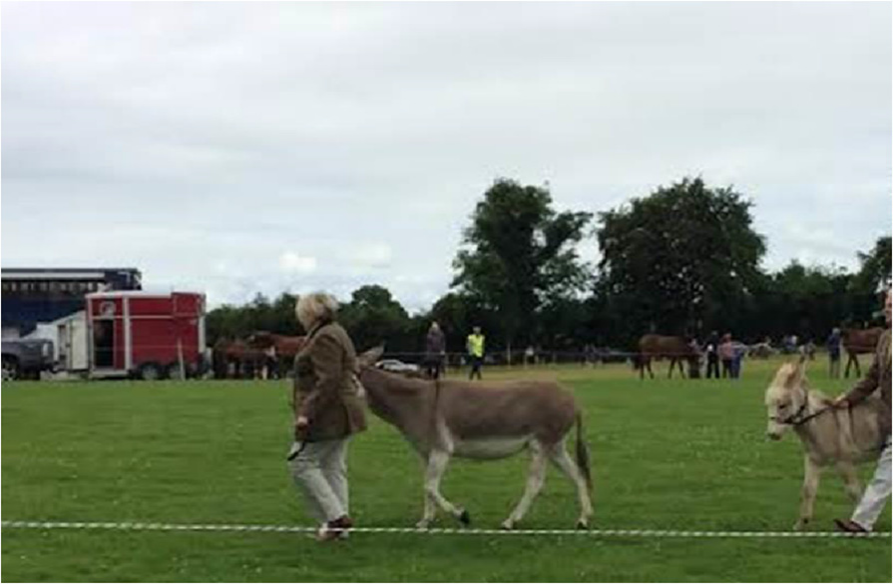
Leading donkeys with horses in the adjacent show ring at Event 2

**Fig. 2:**
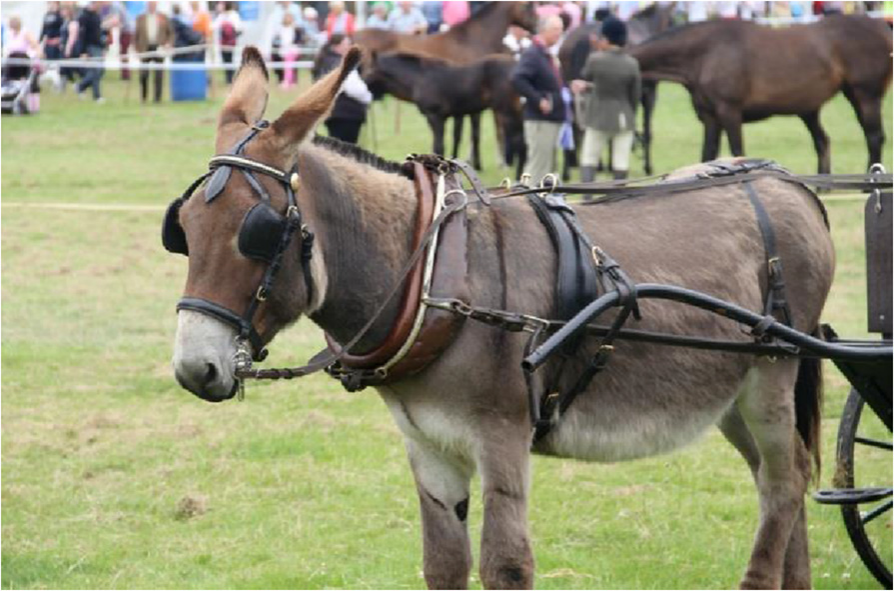
A driving donkey with horses in the adjacent show ring at Event 3

Horses and donkeys were contained in the same areas at seven events while waiting for their classes either in stables or horseboxes parked side-by-side and twice observed in the same horsebox. Show rings were placed immediately adjacent to each other at three events, so contact between horses and donkeys was easily made (see Figs. 1 and 2). At six events, there was sharing of grooming equipment, feed/water buckets, and rugs between donkeys and horses.

One event (event 8) stood in contrast to the others with regard to biosecurity measures: strictly enforced pre-registration requirements; entrance/exit controls; a veterinary presence on-site; checking of documents and vaccination details; and examination of equidae for clinical status on arrival.

#### Compliance with legislation regarding identification and premises registration

The organizers of seven events stated that they required passports to be provided on the day, and at three of those events, equine premises registration was stated as being a requirement to make an entry to an equestrian class. Two events did not require any passport or other identification information for the equines pre-entered for classes and one organiser stated that “some horses were hired for the day and so the competitor making the entry might not have access to that information”. Three events did not require passport and microchip information for donkeys as these were regarded as “livestock” not equines. At eight out of nine events, even when equine passports and microchips were required for pre-registration there was no verification of these documents or details on the day by event organizers or their representatives.

#### Evidence of clinical signs of equine contagious disease

**Fig. 3:**
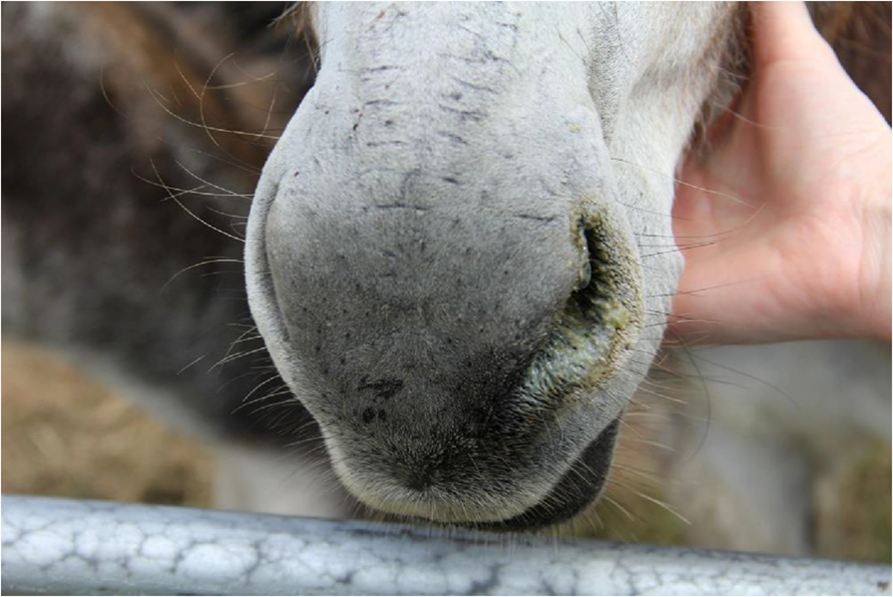
Photograph of a donkey showing evidence of nasal discharge at Event 4

**Fig. 4:**
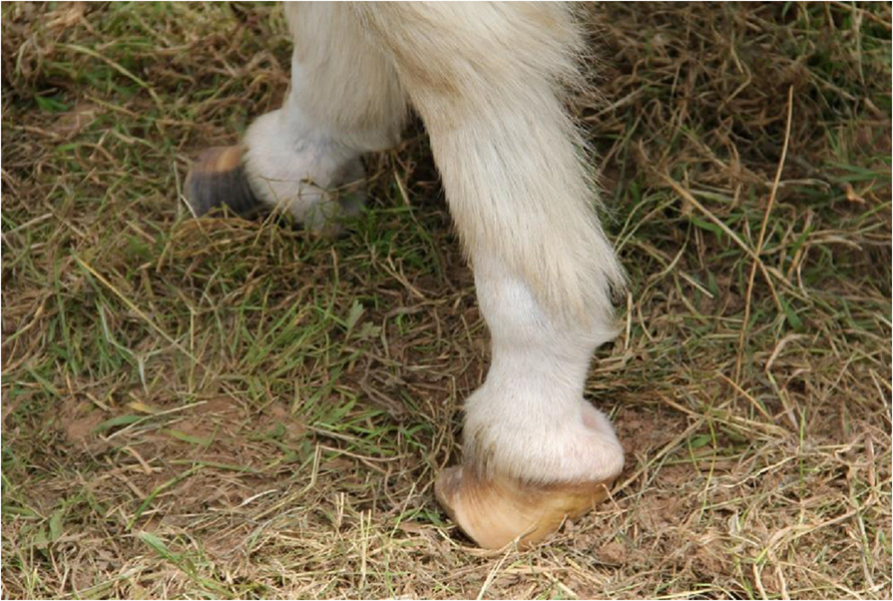
Photograph showing evidence of poor donkey hoof care at Event 4

The animals at all but one event appeared in good health and ideal to fat body condition (Body Condition Scores of 3 and 4 respectively). At event 4 three donkeys were obese (BCS 5/5); poor coats, skin lesions, overgrown hooves, nasal discharge, and evidence of scour in two animals were also identified (see Figs. 3 and 4).

#### Territory of origin

Details of the territory of origin of persons exhibiting horses, ponies or donkeys at one event are presented in Table 2. The place of origin (and identity) of equestrian exhibitors at the other eight events studied could not be verified as pre-registration was not mandatory for all classes and entry controls were not in-place or enforced. However organizers stated that exhibitors were from the island of Ireland.

**Table 2:** The number of exhibitors of equids and their territory of origin at Event 8

**Event 8**	
**Travelled From:**	**Number of event entrants**
Republic of Ireland	820
Northern Ireland	222
Great Britain	15
Continental Europe	13
North America	4

### Donkey exhibitor views

#### Compliance with legislation regarding equidae

Most of the DKs interviewed said that they had passports and microchips for all of their donkeys, but there was no evidence presented to the interviewer at any event to this effect. Two DKs said that donkeys were “not required to have microchips or passports”.

#### Reasons for breeding and keeping donkeys

Only six out of 22 keepers of donkeys (DKs) interviewed said that they still bred their donkeys. Most reported that they kept their donkeys for showing, the next most popular reason for keeping donkeys was driving (Fig. 1), followed by donkey derby racing and, finally, one owner claimed to do agility classes with her donkeys. One DK opined that people kept donkeys for 3 reasons: “nostalgia, as a companion for a horse, or they really love donkeys”. Others expressed the view that people procured them as “lawnmowers” and “companions” and as “livestock units for subsidy payments” or a potential way to “make a quick few quid”. Five DKs kept horses alongside their donkeys and found they “got along quite well”.

#### Procurement of donkeys

**Fig. 5:**
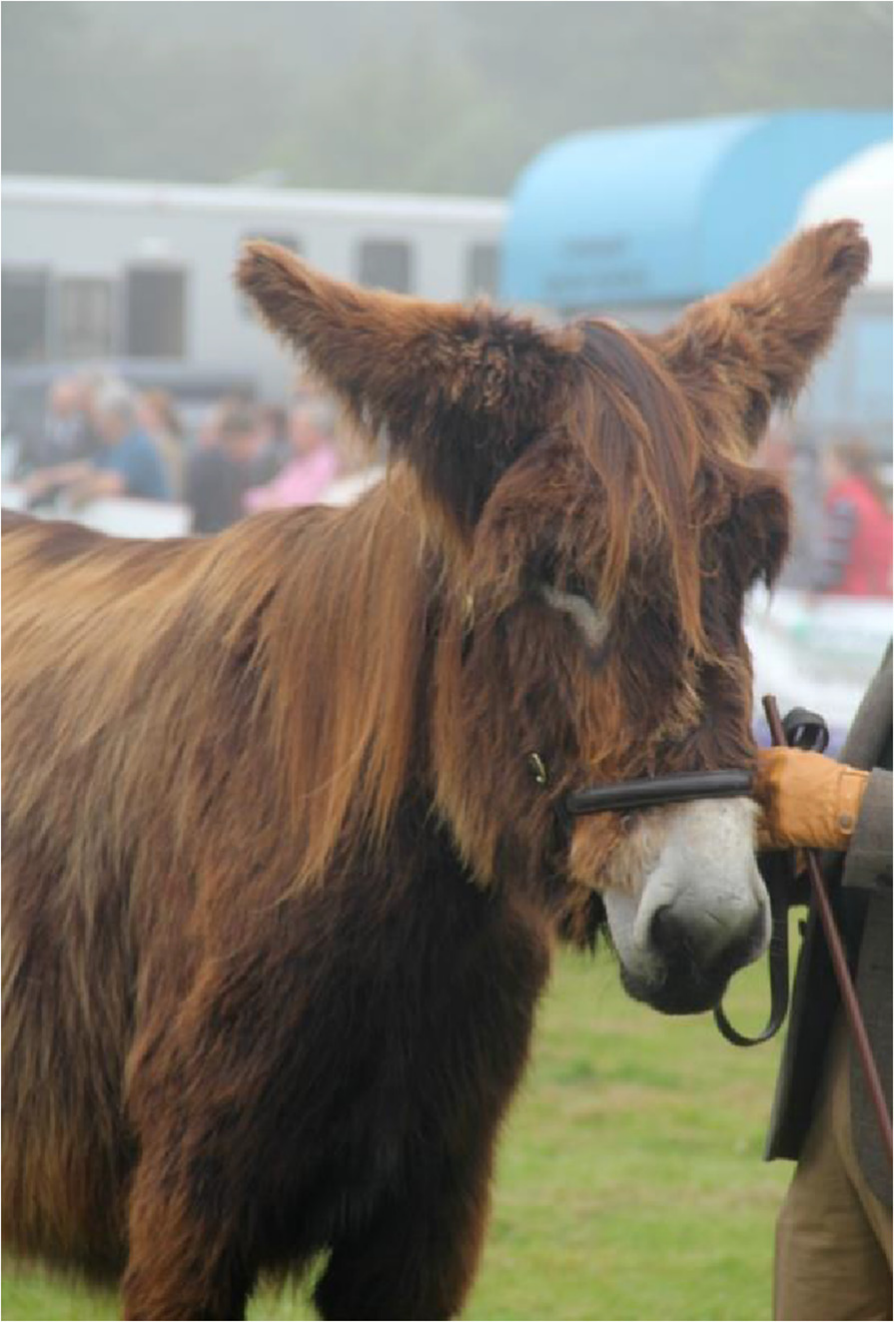
Imported Poitou French breed donkey photographed at Event 3

Some DKs had imported donkeys from Spain and France (see Fig. 5) into Ireland and subsequently moved them around the island exhibiting the different breeds of donkey.

#### Routine husbandry of donkeys

At one event, where clinical signs of disease were observed in donkeys, two exhibitors (of diseased donkeys) said that “donkeys do not need any health care”. Other DKs interviewed said they took good care of their donkeys and ensured that their vaccinations, de-worming, farriery and dentistry were kept up to date.

### Equine exhibitor views

**Fig. 6:**
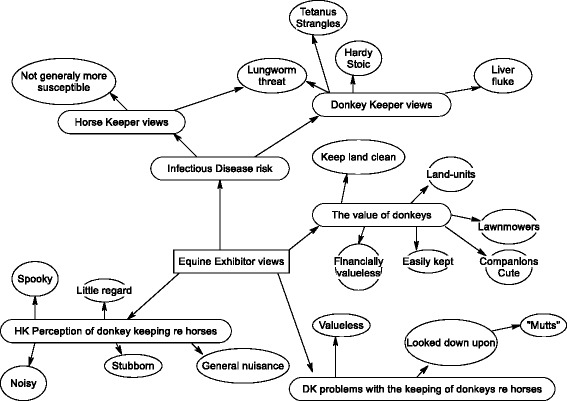
A graphic illustration of themes as articulated by equine exhibitors at mixed equestrian events. Global themes are in rectangular text boxes with straight edges, organizing themes in rectangular text boxes with rounded edges, and basic themes in oval text boxes

Within this overarching or global theme can be found several organizing themes, illustrated graphically in Fig. 6:

1. That related to how the keepers of donkeys (DKs) perceive donkeys as a disease risk
2. That related to how the keepers of horses (HKs) perceive donkeys as a disease risk
3. That related to other risks or problems associated with the keeping of donkeys
4. The general perception of donkeys by HKs
5. Issues related to the value of donkeys

- DK opinion of the donkey as a source of contagious equine disease
  As a basic theme, all but one donkey owner said that they did not see transmissible disease in the donkey: on closer questioning most felt that the donkey was more “hardy” or more “stoic” rather than more ‘resistant’. A second basic theme related to the greater awareness of the potential for parasitic disease most notably lungworm (nematode strongyles) and liver damage due to fluke infestation (trematodes), and on occasion tetanus (non-contagious bacterial infection). As a balancing basic theme the view was expressed that donkeys might ‘clean-up’ rough pasture reducing the incidence of certain diseases for example those transmitted by ticks such as ‘Red-water’ (Babesia) or by consuming parasite eggs.
- HK opinion of the donkey as a source of contagious equine disease
  The core basic theme identified was that most of the HKs interviewed did not view donkeys as a reservoir for disease and thus had no issue with them being kept with their horses. Only one HK said they would not share grazing with a donkey due to concerns about lungworm. One HK questioned said she knew donkeys could get ‘strangles’ (*Streptococcus equi* var *equi* contagious bacterial disease) although she had never witnessed it.
- Problems with the keeping of donkeys
  A key basic theme identified was the negative perception of the keeping of donkeys: multiple DKs opined that HKs often “looked down upon them” for owning donkeys as an inferior animal and laughed at the idea of “showing donkeys”. It was said that it was often the ‘fly-by-nights’ (recent entrants to the competitive equestrian scene) that were the most judgmental of DKs, they opined. It was said that the donkey was considered the “poor man’s horse” and that often HKs considered donkeys to be “mutts” and not to have any useful purpose. One judge (of a donkey showing class) felt that HKs’ main complaint (apart from lungworm) was that horses are often spooked by donkeys and this was a basic theme echoed by many DKs: donkeys were perceived to be a ‘spooky’, noisy, stubborn, ‘eat horses’ tails’ and a general nuisance.
- Actual perception of donkeys by HKs
  In questioning HKs at the events, the key basic themes identified were that the majority had either not considered or were not concerned about donkeys (as reservoirs of equine infectious disease) either specifically at the equestrian events or in general. None of the HKs questioned seemed to mind donkeys being present at the shows and didn’t take much notice of them being there. On closer questioning, themes echoing the views of DKs emerged: horses could be “easily spooked by donkeys”; often due to the “length of their ears” and sometimes due to the “noise they make”. Although they saw no use for donkeys, many thought they were “cute” and “no threat to their horses”.
- The value of donkeys
  An example of a recurring basic theme, was the keeping of donkeys as “land units” to claim on subsidies. Donkeys were generally regarded as “easy to keep” but “valueless” in themselves and thus often meriting little care. They might also kept by some as “companions” or “lawnmowers”.

## Discussion

Apparent compliance with the identification of equidae legislation was quite high among the keepers of donkeys (DKs) interviewed as exhibitors at equestrian events: the majority reported having passports for their donkeys and having had them micro-chipped. However, no passports were offered or made available for examination at any time during the study. Two DKs stated, however, that the law in Ireland did not require passports and micro-chipping for donkeys as it did for horses. The fact that these same two DKs also believed that donkeys did not require other routine health care provisions either, indicates a lack of awareness of basic healthcare and maintenance requirements of donkeys and lack of awareness of the relevant legislation regarding the identification of donkeys; this view was not replicated in conversation with other DKs. The majority of DKs reported that they kept their donkeys up to date on vaccinations, with appropriate de-worming, farriery, and dentistry care. However, no evidence beyond the healthy appearance of the donkeys was available and this view may be indicative of only the sub-set of persons exhibiting donkeys rather than the general donkey-keeping population.

At eight out of nine shows visited across the island of Ireland, no credible control or bio-security measures were in place or enforced that might prevent the spread of contagious disease. Passports and microchip numbers were required to register for most but not all of the events visited. Onsite however, the documentation was not checked and anyone with a horsebox was let onto the fairgrounds. Veterinary examination of equines for identification or clinical status upon entering the show-grounds was performed at only one of nine events, and thus a sick donkey or horse might easily enter any of the other premises with resultant risk to other equines. In fact, donkeys in poor health were seen at one of the events visited in this study. The uncontrolled mixing of un-examined donkeys and horses from unknown backgrounds at these events is the perfect medium for disease transmission. Endemic diseases such as respiratory and intestinal viruses and bacteria, and fungal skin infections can spread with ease from infected horses, ponies or donkeys in close contact with other equidae or where equipment is shared. In Ireland, the greatest threat to the horse population from sub-clinically infected donkeys is arguably lungworm as donkeys are largely asymptomatic and can act as a reservoir for the disease subsequently contracted by horses with clinical consequences. This transmission is unlikely to occur at equine events where horses and donkeys are not grazing for long periods together but may be an issue on the home premises. Other endemic transmissible equine diseases have similar clinical presentations in donkeys and horses although there is a perception as evidenced in this study that donkeys (as compared to horses) are more stoic, show fewer signs of disease and might thus act as reservoirs of same.

However, spread of contagious disease at unregulated equestrian events is particularly critical where donkeys (as for other equidae) are present that have been imported, as these may be asymptomatic carriers of a number of important exotic diseases. Some of the donkeys observed in this study had been imported from Spain and France and moved around Ireland to show at different events. Concern has been raised previously by the authors as to the lack of a comprehensive system recording the movement of equidae within the Tripartite Agreement Zone of Ireland, the UK and France [11]. At one event attended there were equines from as far away as North America and there can be no guarantee, despite the excellent biosecurity measures in place, that animals incubating infectious disease and thus capable of disease transmission were not present.

For many exotic contagious equine diseases, donkeys are more likely than horses to be asymptomatic and are thus potential sources of infection to horses. If any such diseases were to be introduced into Ireland through the importation of sub-clinically infected donkeys/horses it could have devastating consequences for the equine industry here. It is also important to note that many diagnostic tests for these diseases have been validated for horses but not for donkeys and thus all results, both positive and negative should be regarded with caution. Similarly, vaccines against equine infectious diseases have only been tested on horses and thus both dosage and efficacy for donkeys is unknown [4].

Donkeys are not poorly perceived by the keepers of horses (HKs) as reservoirs of disease. It seems that while many HKs would not keep donkeys as they perceive them to have no real function or value, they also perceive them as benign and no great risk to their horses beyond a nuisance value. In fact it appears that both donkey keepers and horse keepers may underestimate the role of the donkey in the transmission of endemic contagious disease in that donkeys are as susceptible as horses to most of our endemic diseases.

## Conclusions

The perception among the keepers of both donkeys and horses interviewed in this study is that donkeys do not pose any significantly increased threat to horses as far as infectious disease transmission is concerned. This is perhaps an underestimation particularly given the findings in this study regarding biosecurity procedures at mixed equestrian events. It seems that leisure and pleasure horse owners do not give much consideration to donkeys at all, thinking them to have “no practical use” and thus not meriting much consideration. The perception (that donkeys are “useless”) rather than any consideration of their role in disease transmission, may act as one driver for the currently low perceived worth of donkeys and thus increase the numbers that must be offered sanctuary by animal welfare organisations. In the authors’ view this possibility merits further consideration.

Equestrian events throughout Ireland pose a real risk for disease spread throughout the equine population due to the lack of and/or enforcement of biosecurity controls. Excepting acting as a reservoir of lungworm infestation which is unlikely to be transmitted at equestrian events of short duration, donkeys do not pose any more risk to the horse population in Ireland than do horses, but their risk as carriers of contagious disease may actually be underestimated. The major threat that donkeys represent to the horse population is donkeys (as per other equines) imported in the absence of proper controls. The enforcement of identification regulations for all equines is fundamental in controlling the movement of equines throughout Europe and managing the spread of equine infectious disease. When biosecurity controls are not in place (or enforced) at equestrian events (particularly in the absence of rigorous importation controls) to actually check passports, verify identification and ensure compliance with the requirements to register equine premises, such events may unwittingly act as the mechanism of spread of endemic and potentially more seriously exotic equine infectious disease.
